# The impact of citrate introduction at UK syringe exchange programmes: a retrospective cohort study in Cheshire and Merseyside, UK

**DOI:** 10.1186/1477-7517-4-21

**Published:** 2007-12-11

**Authors:** Caryl M Beynon, Jim McVeigh, Martin Chandler, Michelle Wareing, Mark A Bellis

**Affiliations:** 1 Centre for Public Health, Faculty of Health and Applied Social Sciences, Liverpool John Moores University, Castle House, North Street, Liverpool, L3 2AY, UK

## Abstract

**Background:**

In 2003, it became legal in the UK for syringe exchange programmes (SEPs) to provide citrate to injecting drug users to solubilise heroin. Little work has been undertaken on the effect of policy change on SEP function. Here, we examine whether the introduction of citrate in Cheshire and Merseyside SEPs has altered the number of heroin/crack injectors accessing SEPs, the frequency at which heroin/crack injectors visited SEPs and the number of syringes dispensed.

**Methods:**

Eleven SEPs in Cheshire and Merseyside commenced citrate provision in 2003. SEP-specific data for the six months before and six months after citrate was introduced were extracted from routine monitoring systems relating to heroin and crack injectors. Analyses compared all individuals attending pre and post citrate and matched analyses only those individuals attending in both periods (defined as 'longitudinal attenders'). Non-parametric tests were used throughout.

**Results:**

Neither new (first seen in either six months period) nor established clients visited SEPs more frequently post citrate. New clients collected significantly less syringes per visit post citrate, than pre citrate (14.5,10.0; z = 1.992, P < 0.05). Matched pair analysis showed that the median number of visits for 'longitudinal attenders' (i.e. those who attended in both pre and post citrate periods) increased from four pre citrate to five post citrate (z = 2.187, P < 0.05) but the number of syringes collected remained unchanged. These changes were not due to seasonal variation or other changes in service configuration.

**Conclusion:**

The introduction of citrate did not negatively affect SEP attendance. 'Longitudinal attenders' visited SEPs more frequently post citrate, providing staff with greater opportunity for intervention and referral. As the number of syringes they collected each visit remained unchanged the total number of clean syringes made available to this group of injectors increased very slightly between the pre and post citrate periods. However, new clients collected significantly less syringes post citrate than pre citrate, possibly due to staff concerns regarding the amount of citrate (and thus syringes) to dispense safely to new clients. These concerns should not be allowed to negatively impact on the number of syringes dispensed.

## Background

Syringe exchange programmes (SEPs) were established in the United Kingdom (UK) in the 1980s in response to the arrival of HIV infection and concerns regarding its transmission through the sharing of injecting paraphernalia. Such policies were driven by a public health perspective which regarded the prevention of the spread of HIV infection to be more important than preventing any potential drug users from injecting [1]. While it was legal to supply clean needles and syringes, supplying a person with any other article which the supplier believed the recipient may use to administer unlawful drugs, or prepare unlawful drugs for administration, remained an illegal activity. In 2002 the Advisory Council on the Misuse of Drugs (ACMD) were asked to consider the harm reduction benefits of drug paraphernalia other than syringes and needles. The following year, the ACMD recommendations were accepted and, on 1^st ^August 2003, it became legal to supply ampoules of water, swabs, utensils for drug preparation (spoons, cups etc.), citrate and filters [2].

The majority of drug users who use SEPs in England to obtain clean injecting equipment are injectors of heroin and the majority of these will inject 'brown' heroin [3]. Brown heroin is sold in poorly soluble base form and most injecting drug users will use an acidifier (for example citric, ascorbic and lactic acids) to chemically convert it to a soluble injectable form [4]. Readily available forms of these acids include fresh and processed lemon juice, vinegar and other household products but injecting such substances have reportedly resulted in infections such as endocarditis (infection of the heart valves) and endopthalmitis (infection of the eyes, [5]).

No acidifier can be considered safe, but citrate is believed to be the safest to use for the preparation of brown heroin [6]. Whilst the provision of citrate sachets is relatively new in the UK its availability in other European countries is reported to have increased the use of SEPs, reduced the use of more dangerous acidifiers, has been popular with injecting drug users and has improved their relationship with SEP staff [7]. Whilst it was hoped that the introduction of citrate sachets would increase both the number of people attending SEPs and the number of visits they made [6], to date this has not been evaluated within the UK. Therefore, here we use an established syringe exchange monitoring system to compare SEP profiles of clients attending in the six months pre citrate and six months post citrate and examine whether introducing citrate has altered: the number of heroin/crack injectors accessing SEPs; the number of times heroin/crack injectors visit SEPs and the number of syringes dispensed per visit.

## Methods

Cheshire and Merseyside has a population of 2,345,077 (4.7% of England) and its drug users are served by 15 SEPs based within drug services (pharmacy based SEPs are also available). Of these, 11 were identified as introducing citrate between May 2003 and October 2004 and consequently were included in the study.

Details of the SEP monitoring system, established in Cheshire and Merseyside in 1991, are reported elsewhere [8]. This well-established monitoring system, using the principles of the National Drug Treatment Monitoring System within England for the collection of data on structured drug treatment service provision [9], enables each syringe transaction to be attributed to a specific individual and service. An individual is identified by their attributor code comprised of their initials, date of birth and gender [8].

Each of the 11 participating SEPs were contacted to identify the exact date that citrate provision commenced. Monitoring data for the six months pre citrate and post citrate were extracted individually for each of the 11 SEPs. Within each six months (i.e. pre citrate and post citrate separately), attributor codes were used to aggregate an individual's transactions into a single client profile for people who had attended SEPs to collect clean syringes. Again, within each six-month period, individuals were identified as either a 'new' client (no previous contact with the SEP) or an 'established' client (previous contact with the SEP between 1991 and the six months in question). Final person-specific pre and/or post citrate profiles for each SEP included age at most recent SEP contact, gender, the clients main injected drug, their number of visits and the median number of syringes collected per visit.

Individuals whose main injected drug was heroin or crack cocaine and who were recorded as having collected clean syringes on at least one occasion during either six month period were included in the analyses. Final SEP-specific profiles for pre and post citrate periods comprised the number of individuals, median number of visits per client and median syringes collected per visit for both new and established clients. In addition to comparing pre and post citrate profiles, individuals who were identified in both pre and post citrate periods were included in matched pair analyses, where an individual's pre citrate profile was compared with their own profile for the post citrate period. For the purpose of this study, individuals included in matched analyses are termed 'longitudinal attenders' because they were recorded in both pre and post citrate periods. It is worth noting, however, that these longitudinal attenders may only have visited a SEP once in each six month period.

Two additional analyses were necessary to assess whether any observed changes pre versus post citrate were related to natural seasonal variation or other changes in service configuration. Therefore all heroin/crack SEP injectors recorded in the post citrate period were matched with their own SEP profiles for the corresponding time period 12 months previously (n = 314 matched pairs) and the median number of visits and syringes collected compared. Finally, pre and post citrate profiles for anabolic steroid injectors were extracted using the same six month pre and six month post citrate protocol detailed for heroin and crack injectors (n = 295 matched pairs). It was hypothesised that, because steroid users do not use an acidifier, there would be no significant difference in the median visit rate or median number of syringes collected pre versus post citrate.

### Statistical analyses

Kolmogorov-Smirnov tests showed that age, the number of visits and the number of syringes collected were all significantly, positively skewed, with the exception of the age of new clients. Non-parametric tests have therefore been used throughout with Wilcoxon sign rank tests used for matched, and Mann Whitney U for unmatched, data. Chi square analyses were used to compare categorical data and all analyses were undertaken using SPSS version 12 [10] or EpiInfo version 6 (for chi square [11]).

## Results

Comparing pre and post citrate periods, there was no significant difference in the age and gender of either new or established SEP clients (Table 1). The median number of syringes collected per visit by new clients significantly decreased from 14.5 syringes pre citrate to 10 syringes post citrate (P < 0.05). All other variables did not differ significantly pre versus post citrate.

**Table 1:** Pre versus post citrate comparisons in 11 syringe exchange programmes in Cheshire and Merseyside, UK

	**Six Month Reporting Period**			
	
	**Established clients**		**New clients**	
	
	**Pre citrate**	**Post citrate**	**Pre citrate**	**Post citrate**
	
	n		n	
Individuals	584	544	258	258
Visits	3485	3734	806	854
Total syringes collected	82101	80074	16947	13059
	%		%	
Male	83.6	84.4	82.6	78.3

	**Median (Interquartile range)**		**Median (Interquartile range)**	

Age	33.72 (29.89, 38.02)	34.39 (30.43, 38.25)	32.46 (27.75, 36.86)	31.97 (27.67, 35.72)
Visits per person	3 (1, 8)	3 (1, 9)	2 (1, 4)	2 (1, 4)
Syringes collected per person	15 (10, 30)	15 (9.25, 27.5)	14.5 (7.88, 25.0)	10 (6.5, 20.0)^1^

^1^Significant at the <0.05 level

Table 2 reports findings from the matched pair analyses for longitudinal attenders of SEPs. The number of syringes collected by heroin/crack injectors did not differ between pre versus post citrate and the median visit rate significantly increased from four visits pre citrate to five visits post citrate. Matched pair analyses comparing the profiles of heroin/crack injectors post citrate with their own corresponding profile 12 months previously showed no difference in the median number of syringes collected per visit, but a significant increase in the median number of visits made per person was observed (P < 0.005). Matched pair analyses comparing steroid injector profiles pre versus post citrate showed no difference in the median number of visits made per client and the median number of clean syringes collected.

**Table 2:** Impact of citrate introduction in 11 syringe exchange programmes in Cheshire and Merseyside, UK

	**Six Month Reporting Period**		
	
	**12 months pre citrate**^1^	**Pre citrate**	**Post citrate**
	
	**Median (Interquartile range)**		
	
	**Established matched pairs (n = 398)**		
Visits per person	--	4	5
	--	(2, 10)	(2, 11)^2^
Syringes collected per person	--	15	15
	--	(10, 30)	(10, 25)

	**Seasonal matched pairs (n = 314**)		

Visits per person	4	--	5
	(2, 9)	--	(2.75, 12.25)^3^
Syringes collected per person	15	--	20
	(10, 30)	--	(10, 30)

	**Steroid matched pairs (n = 295)**		

Visits per person	--	1	1
	--	(1, 2)	(1, 2)
Syringes collected per person	--	30	30
	--	(20, 36)	(20, 40)

^1^12 months pre citrate' corresponds to the post citrate period, 12 months previously and has been used as a control to asses the possibility that any differences between pre and post citrate were due to natural seasonal variations.

^2^Significant at the <0.05 level

^3^Significant at the <0.005 level

## Discussion

Worldwide there are an estimated 13.2 million injecting drug users [12]. In addition to the high risk of overdose amongst this group [13,14], drug users who choose to inject are particularly vulnerable to a range of infectious diseases, including viral infections such as HIV and hepatitis, and bacterial infections such as Group A Streptococci and *Staphlococcus aureus*, resulting in considerable levels of morbidity and mortality [15]. Growing concern regarding these injecting-related health problems is reflected in recent changes in the UK law, which in 2003, sanctioned the dispensing of injecting paraphernalia reported to have harm reduction benefits, in addition to the provision of clean needles and syringes. Under these amendments, it became legal in the UK to provide citrate to injecting drug users, a substance shown to be an appropriate means by which to convert street heroin into a soluble form [3].

It was envisaged that the introduction of citrate would increase both the number of injectors attending SEPs and the number of visits each person made [6]. The recently updated guidance on the commissioning and provision of treatment for adult drug users [16] highlights the need for the reinvigoration of harm reduction activities across all treatment tiers (drug-related interventions in England and Wales fall into a tier structure that reflects the increasing intensity of the interventions). Increasing the number of individuals in contact with SEPs and the frequency of their engagement are positive public health indicators for harm reduction development. Analysis of SEP monitoring data showed no increase between pre and post citrate periods in the number of established or new clients. Importantly however, there was no significant decrease either, showing that the introduction of citrate had not negatively affected attendance.

Monitoring data also showed no significant increase in the frequency with which heroin/crack injectors attended SEPs following citrate's introduction. The median number of visits made by established clients was three in both pre and post citrate periods while new clients made, on average, two visits within each of the two six month periods. However, matched pair analyses of longitudinal attenders of SEPs, comparing an individual's post citrate profile with their own behaviour pre citrate, showed that this cohort of injectors made significantly more visits post citrate (median = 5) than pre citrate (median = 4, P < 0.05). Further to this, matched pair analyses showed the median visits per person post citrate (median = 5) was significantly greater than the median visits for the corresponding six months in the previous year (median = 4, P < 0.005), for those injectors who were recorded in these two six month periods. We can therefore discount the possibility that the increase in visit rate between pre and post citrate was due to seasonal variation because the increased visit rate following the introduction of citrate occurred across years as well as within the year. Additionally, again using matched pair analysis, we observed no difference in the median number of visits pre and post citrate for steroid injectors (median number of visits being one in both the pre and post citrate periods). Steroid users do not use an acidifier so their behaviour should not be affected by the introduction of citrate. That no change in the behaviour of steroid injectors was observed supports the conclusion that the increased visit rate post citrate of heroin/crack injectors who attended SEPs in both pre and post citrate periods was due to the introduction of citrate. It is important to note, however, that the legal changes that permitted the distribution of citrate also sanctioned the distribution of other injecting paraphernalia (for example, spoons and water), although the distribution of other paraphernalia in SEPs in Cheshire and Merseyside occurred less consistently than the introduction of citrate. Despite this, it is possible that the distribution of other injecting paraphernalia also affected the behaviour of SEP attenders within this geographical area.

From these findings, we can conclude that the introduction of citrate did not encourage more clients to contact SEPs to collect clean injecting equipment in the first six months of its introduction, nor can we conclude that its introduction negatively affected attendance. Furthermore, we cannot conclude that overall, people visited SEPs more frequently following the introduction of citrate but that its introduction has encouraged longitudinal attenders of SEPs (i.e. those who were recorded in both the pre and post citrate six month periods) to visit more frequently. Therefore, at SEPs included in this study, the introduction of citrate has resulted in a change in service use amongst certain SEP clients, with less impact on those injectors who visit SEPs infrequently. Any increase in visit frequency should be welcomed as it provides SEP staff greater opportunity to engage with injectors to discuss a range of harm reduction measures and, where appropriate, to refer into other services. It is not clear from this study whether further changes will be observed once information about the availability of citrate at SEPs becomes universal amongst the injecting community.

With respect to the number of syringes collected per visit, no difference was evident pre versus post citrate for established SEP clients. Matched pair analysis showed comparable findings. Similarly, no difference was observed in the number of syringes collected for heroin/crack injectors post citrate compared to the corresponding six month period in the previous year or for steroid injectors pre versus post citrate. Established SEP clients are therefore continuing to receive the same number of syringes per visit and, presumably, sufficient citrate for the number of syringes dispensed. Injectors who are classified as 'longitudinal attenders' for the purpose of this study, are thus attending SEPs more frequently post citrate but collecting the same number of syringes per visit, increasing, very slightly, the total number of syringes dispensed to this cohort of injectors from 71,495 in the pre citrate six month period to 71,743 in the post citrate six month period (data not shown). In light of evidence to suggest that clean syringes are used in only 25% of injections [17], from a public health perspective, any increase in syringe provision is welcome.

Despite the benefits of citrate over other acidifiers [7], all may result in vein damage and the smallest possible amount is recommended to solubilise heroin. Consultation with injecting drug users resulted in the current practice of dispensing citrate in 100 mg sachets [6]. This amount was deemed sufficient to dissolve the £20 of heroin normally prepared and because packaging a smaller amount would be unfeasible. Injectors liked the idea of single use sachets which were also deemed to decrease the risk of contamination from sharing whilst encouraging hygienic injecting techniques. Monitoring data showed that SEP staff dispensed significantly less syringes to new clients per visit post citrate (median = 10 per visit) than pre citrate (median = 14.5 per visit, P < 0.05). While it is important that SEP staff are aware of the potential harm excess citrate may cause, fears regarding the dispensation of too much citrate to new clients must not be allowed to impact negatively on the number of clean syringes dispensed.

## Conclusion

While citrate may be the safest acidifier for drug users to prepare heroin for injection, hopes that its introduction would increase the number of injectors accessing SEPs were not supported by this study. Importantly, however, the introduction of citrate did not negatively affect attendance either, indicating that citrate can be added to the spectrum of interventions offered by SEPs without any apparent negative consequences. Furthermore, injectors who already attended SEPs relatively frequently on a longitudinal basis (i.e. in both pre and post citrate periods), attended SEPs more frequently, providing evidence to support a positive change in service use among these particular individuals. Greater levels of engagement provide increased opportunities for interaction between the injecting drug user and the practitioner. This can facilitate a range of harm reduction interventions relating to the prevention of blood borne infections and improvements in injecting techniques. In addition, increased contact rates can provide opportunities for appropriate referral to both specific drug-related interventions and generic health and welfare support. Furthermore, the increased number of syringe exchange visits, without a reduction in the number of syringes provided at each visit, within this client group, has slightly increased the number of clean syringes in circulation. Increasing the number of clean syringes distributed, to enable the use of sterile equipment for each injection, should remain a public health target and developments to facilitate this should be supported. Staff concerns regarding the amount of citrate to dispense to new clients must not impact on the number of syringes given out. It is not clear from this study whether further changes have been observed at SEPs once information about the availability of citrate became ubiquitous among drug injectors. Finally, this study demonstrates the value of utilising routinely collected monitoring data to assess the impact of harm reduction interventions, with further analyses planned to evaluate the longer-term impact of citrate provision at SEPs.

## Competing interests

The author(s) declare that they have no competing interests.

## Authors' contributions

CMB carried out data extraction, performed the statistical analyses and wrote the manuscript. JM conceived of the study, participated in its design and was involved in writing the manuscript. MC coordinated acquisition of data and assisted in writing the manuscript. MW participated in writing the manuscript. MAB provided assistance with the statistical analyses and interpretation and helped to revise the manuscript. All authors read and approved the final manuscript.
